# Diagnostic yield of next-generation sequencing in suspect primary immunodeficiencies diseases: a systematic review and meta-analysis

**DOI:** 10.1007/s10238-024-01392-2

**Published:** 2024-06-18

**Authors:** Yingying Chen, Dongrui Li, Jiawen Yin, Jinglin Xiong, Min Xu, Qing Qi, Wenlin Yang

**Affiliations:** 1 Department of Dermatology, The Second Affiliated Hospital of Guangzhou Medical University, Guangzhou, 510260 China; 2 The First Clinical College of Guangzhou Medical University, Guangzhou, 510180 China

**Keywords:** Diagnostic yield, Next-generation sequencing, Primary immunodeficiencies diseases, Meta-analysis

## Abstract

To determine the diagnostic yield of Next-generation sequencing (NGS) in suspect Primary Immunodeficiencies Diseases (PIDs). This systematic review was conducted following PRISMA criteria. Searching Pubmed and Web of Science databases, the following keywords were used in the search: (“Next-generation sequencing”) OR “whole exome sequencing” OR “whole genome sequencing”) AND (“primary immunodeficiency disease” OR “PIDs”). We used STARD items to assess the risk of bias in the included studies. The meta-analysis included 29 studies with 5847 patients, revealing a pooled positive detection rate of 42% (95% CI 0.29–0.54, *P* < 0.001) for NGS in suspected PID cases. Subgroup analyses based on family history demonstrated a higher detection rate of 58% (95% CI 0.43–0.71) in patients with a family history compared to 33% (95% CI 0.21–0.46) in those without (*P* < 0.001). Stratification by disease types showed varied detection rates, with Severe Combined Immunodeficiency leading at 58% (*P* < 0.001). Among 253 PID-related genes, RAG1, ATM, BTK, and others constituted major contributors, with 34 genes not included in the 2022 IUIS gene list. The application of NGS in suspected PID patients can provide significant diagnostic results, especially in patients with a family history. Meanwhile, NGS performs excellently in accurately diagnosing disease types, and early identification of disease types can benefit patients in treatment.

## Introduction

Primary Immunodeficiency Diseases (PIDs), also recognized as Inborn Errors of Immunity (IEI), constitute a diverse set of monogenic disorders that impact the immune system. The clinical spectrum of PIDs encompasses various phenotypes, including infections, autoinflammation, autoimmunity, allergies, malignancies, and more [9]. Presently, over 430 genes associated with PIDs have been identified, predominantly inherited in a monogenic manner. The list of disease-causing genes continues to expand with ongoing discoveries. The International Union of Immunological Societies (IUIS) classifies IEI into 10 categories, addressing diseases with overlapping phenotypes (citation). These categories range from Combined Immunodeficiencies to Congenital Immune Deficiencies (citation). Given the broad clinical spectrum exhibited by PIDs, achieving an accurate diagnosis based solely on clinical manifestations poses a significant challenge. Hence, there is an urgent need in clinical practice for methods that are safe, rapid, and accurate for testing and diagnosing PIDs.

Before the advent of advanced technologies, Sanger sequencing was the primary method employed in clinical practice. However, its limitations became evident in the context of PIDs due to genetic pleiotropy and heterogeneity. Sanger sequencing, being time-consuming, labor-intensive, inefficient, and relatively expensive, faced challenges in identifying disease-causing genes [19, 23]. The emergence of next-generation high-throughput sequencing technologies marks a transformative era in PID diagnosis. Next-generation sequencing (NGS) has not only expanded the known gene list associated with PIDs but has also introduced a faster and more cost-effective means of evaluating the genome. Particularly in cases lacking clear candidate genes, NGS becomes indispensable. NGS encompasses various techniques, including Targeted Gene Panel (TGP) sequencing, Whole Exome Sequencing (WES), and Whole Genome Sequencing (WGS) [39].

TGP is a method focusing on a limited set of genes relevant to specific phenotypes or disease groups and is used for patients with well-defined clinical phenotypes [16, 31]. WES, targeting the exome region, can identify genetic variations associated with classical or atypical phenotypes, expanding the understanding of new phenotypes and genes. Diagnostic yields from WES vary widely (15–79%) depending on patient types and clinical phenotypes (citation). WGS, covering the entire genome, provides better resolution for detecting copy number variations (CNVs) and structural variations. However, it introduces the challenge of interpreting a substantial number of variants of uncertain significance, especially in non-coding regions [10, 20].

As technology advances and costs decrease, NGS plays an increasingly vital role in primary PID diagnosis. Nevertheless, literature reports indicate significant variation in the diagnostic rates of NGS in PIDs. Hence, there is a crucial need to comprehensively evaluate the technical performance and diagnostic efficacy of NGS in PID patients across diverse populations and diseases. This meta-analysis aims to fulfill this need through a comprehensive evaluation.

## Method

### Search strategy

**Fig. 1:**
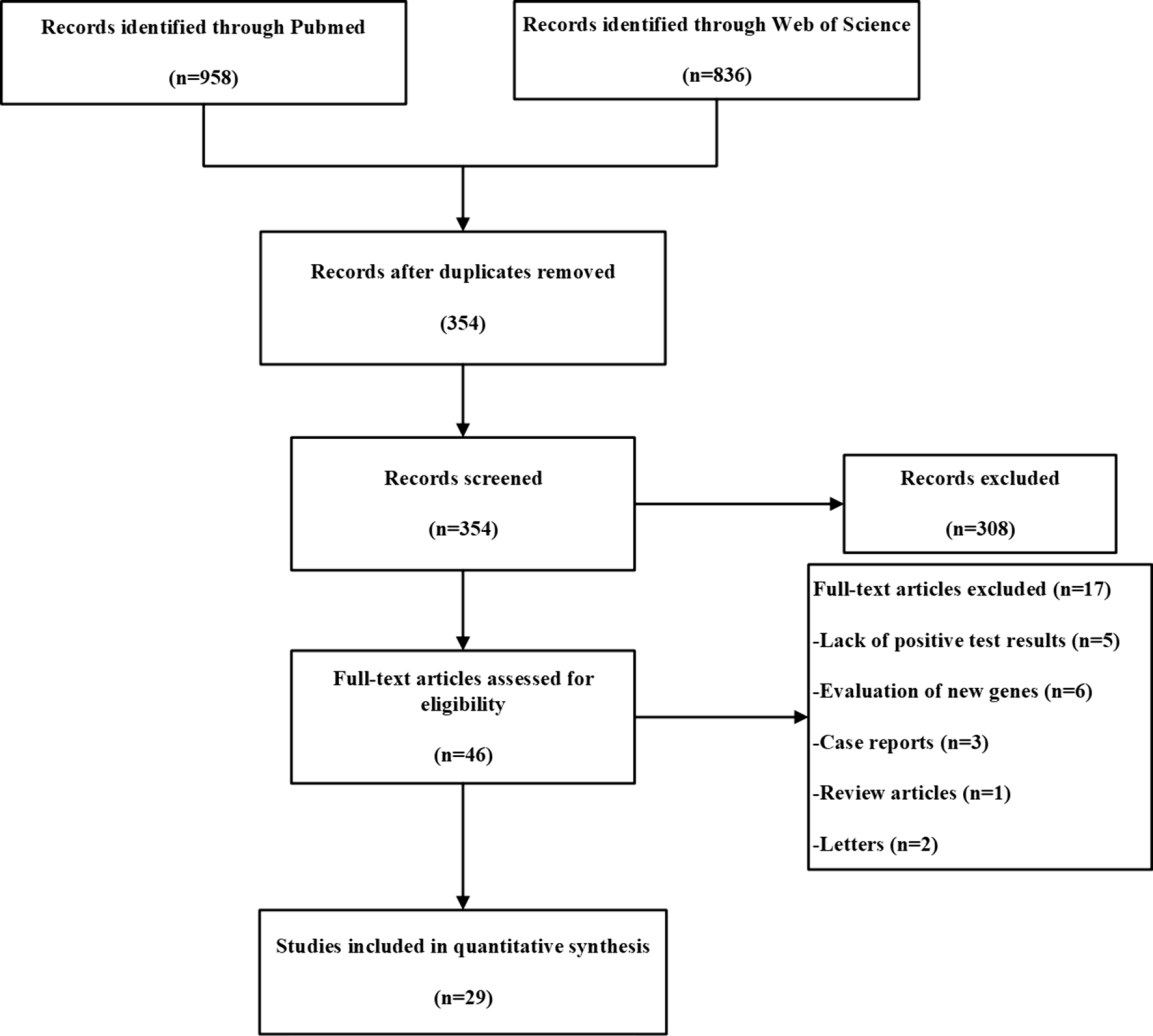
PRISMA flow diagram

A comprehensive search for pertinent studies was systematically conducted in the Pubmed and Web of Science databases up to November 1st, 2023. The search strategy employed the following Boolean operators: ("next-generation sequencing" OR "whole exome sequencing" OR "whole genome sequencing") AND ("primary immunodeficiency disease" OR "PIDs"). A total of 958 studies were retrieved from Pubmed, and 836 studies were identified from Web of Science. No additional pertinent studies were discerned in the bibliographies of the encompassed studies. A transparent depiction of the search and literature screening process is presented in Fig. 1.

### Literature selection

In adherence to predefined inclusion and exclusion criteria, studies were meticulously selected for the meta-analysis. Inclusion criteria stipulated: (1) a study population exhibiting clinically evident manifestations of PIDs; (2) incorporation of genetic sequencing methodologies and provision of pertinent genetic testing information; (3) explicit reporting of both the total number of patients and the number of positive patients detected; and (4) documentation in the English language. Exclusion criteria comprised: (1) studies exclusively focused on the detection of copy number variations (CNVs); and (2) exclusion of duplicate publications, conference proceedings, case reports, reviews, and unpublished studies.

### Data extraction and quality assessment

A rigorous evaluation of potentially relevant articles commenced with an independent screening of titles and abstracts by three authors. Subsequent data extraction, in concordance with predefined inclusion criteria, encompassed key parameters such as the first author, publication year, detection method, total number of patients, number of positive patients, and genetic locus information. The assessment of the risk of bias in the included studies adhered to the Standards for Reporting of Diagnostic Accuracy Studies (STARD) criteria. Any discrepancies in the extraction and assessment processes were resolved through meticulous discussion. In instances where consensus remained elusive, a third reviewer assumed the role of an arbitrator.

### Analytical procedures

All statistical analyses were conducted utilizing the meta package in Stata 14. A meta-analysis of the gene testing detection rate for PIDs was undertaken utilizing a single-group rate approach. Effect size (ES) and a 95% confidence interval (CI) were computed employing a random-effects model. Subgroup analyses discerned the detection rate of gene testing for distinct populations and varied diseases. Heterogeneity was quantified employing the chi-square Q test and I^2^ test. A fixed-effects model was employed when I^2^ was less than 50% or the *p* value of the Q test exceeded 0.05. Conversely, in instances where I^2^ surpassed 50% or the *p* value of the Q test fell below 0.05, a random-effects model was applied.

## Results

### Characteristics of included studies

A total of 29 studies [1–5, 11, 14, 18, 21, 24, 27, 30, 32, 37, 38, 6–8, 12, 13, 15, 22, 25, 26, 28, 29, 36, 45, 46], encompassing 5847 patients, were included in this meta-analysis. Among these, 10 studies conducted familial analyses across various centers and countries. The studies employed diverse sequencing methodologies, including whole-exome sequencing (WES), Whole Genome Sequencing (WGS), targeted DNA sequencing, and clinical exome sequencing (CES). Detailed characteristics of the included studies are summarized in Table 1.

**Table 1:** Characteristics of included studies

Author	Country	Clinically diagnosed as PIDs (Yes/No)	Included cases (N)	Positive diagnosis on ES (N)	Sequencing approach	Novel variants (N)
Baran Erman et al. (2019)	Turkish	Yes	8	5	NGS	3
Francesc Rudilla et al. [30]	Spain	Yes	61	19	CES	12
Adiratna Mat Ripen et al. [29]	Malaysia	Yes	30	14	WES	2
Platt et al. (2020)	NA	No	878	498	WES	28
Chikako Kamae et al. [18]	Japan	Yes	11	6	WES	3
Asgardoon et al. [7]	Iran	Yes	83	27	WES	NA
Peer Arts et al. [5]	International	No	254	72	WES	1
Arun Kumar et al. (2021)	Indian	Yes	229	97	NGS	40
Hassan Abolhassani et al. (2017)	Iranian	Yes	243	176	Targeted DNA sequencing	NA
Waleed Al-Herz et al. [2]	Kuwait	Yes	206	154	WES	5
Hamoud Al-Mousa et al. (2015)	Saudi Arabia	Yes	139	35	NGS	44
Baran Erman et al (2017)	Turkish	NO	19	6	NGS	4
Hui Yu et al. [45]	International	Yes	20	14	NGS	2
Nijman et al (2013)	International	Yes	26	4	NGS	NA
William Rae et al. [27]	UK	Yes	27	13	NGS	NA
Kim Elsink et al. [12]	Netherlands	Yes	165	25	NGS	NA
Patrick Maffucci et al. [21]	USA	Yes	50	15	WES	NA
Tasuku Suzuki et al. (2017)	Japan	Yes	35	5	WES	NA
Mitra Tafakori et al. (2019)	International	Yes	48	35	WES	NA
Zahra Shahbaz et al. (2019)	Iranian	Yes	62	50	WES	27
Clair Engelbrecht et al. [13]	southern Africa	Yes	80	24	WES	NA
Sara Bohnstedt et al. (2022)	Denmark	No	94	14	WES	5
Cristina Cifald et al. (2019)	Italian	Yes	105	30	NGS	18
Tsubasa Okano et al. [25]	Japan	Yes	136	36	WES	NA
Alessandro Borghesi et al. [8]	Switzerland	No	176	41	WES	NA
Jahnavi Alur et al. (2019)	NIIH	Yes	57	49	Sanger T-NGS	NA
Jinqiao Sun et al. [36]	China	No	2392	38	NGS	NA
Tianwen Zhu et al. [46]	China	Yes	72	24	NGS	NA
Amit Rawat et al. [28]	USA	Yes	121	77	NGS	NA

*NIIH* National Institute of Immunohaematology

### Primary outcome analysis

**Fig. 2:**
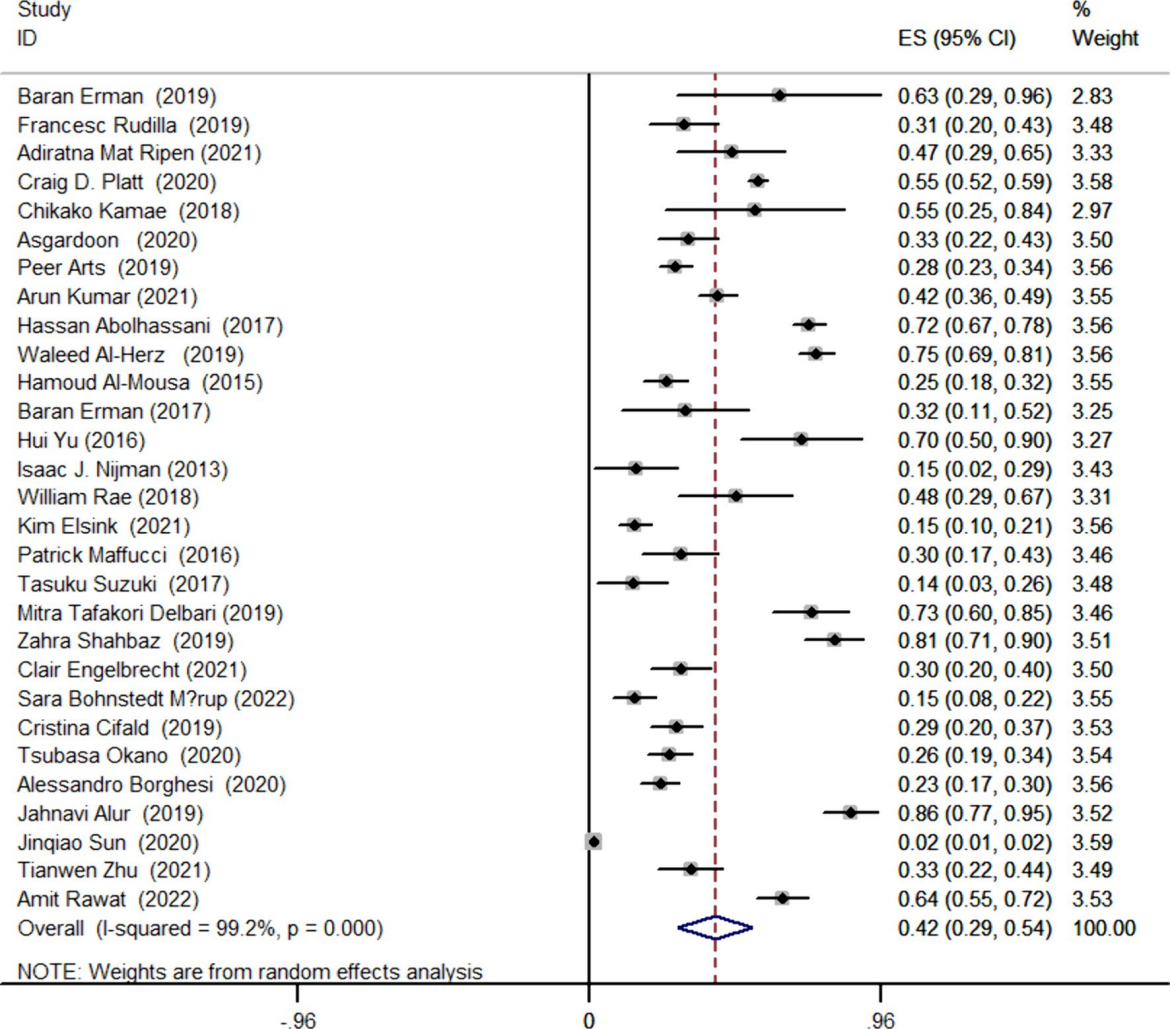
The overall physical examination rate of primary immunodeficiency diseases

**Fig. 3:**
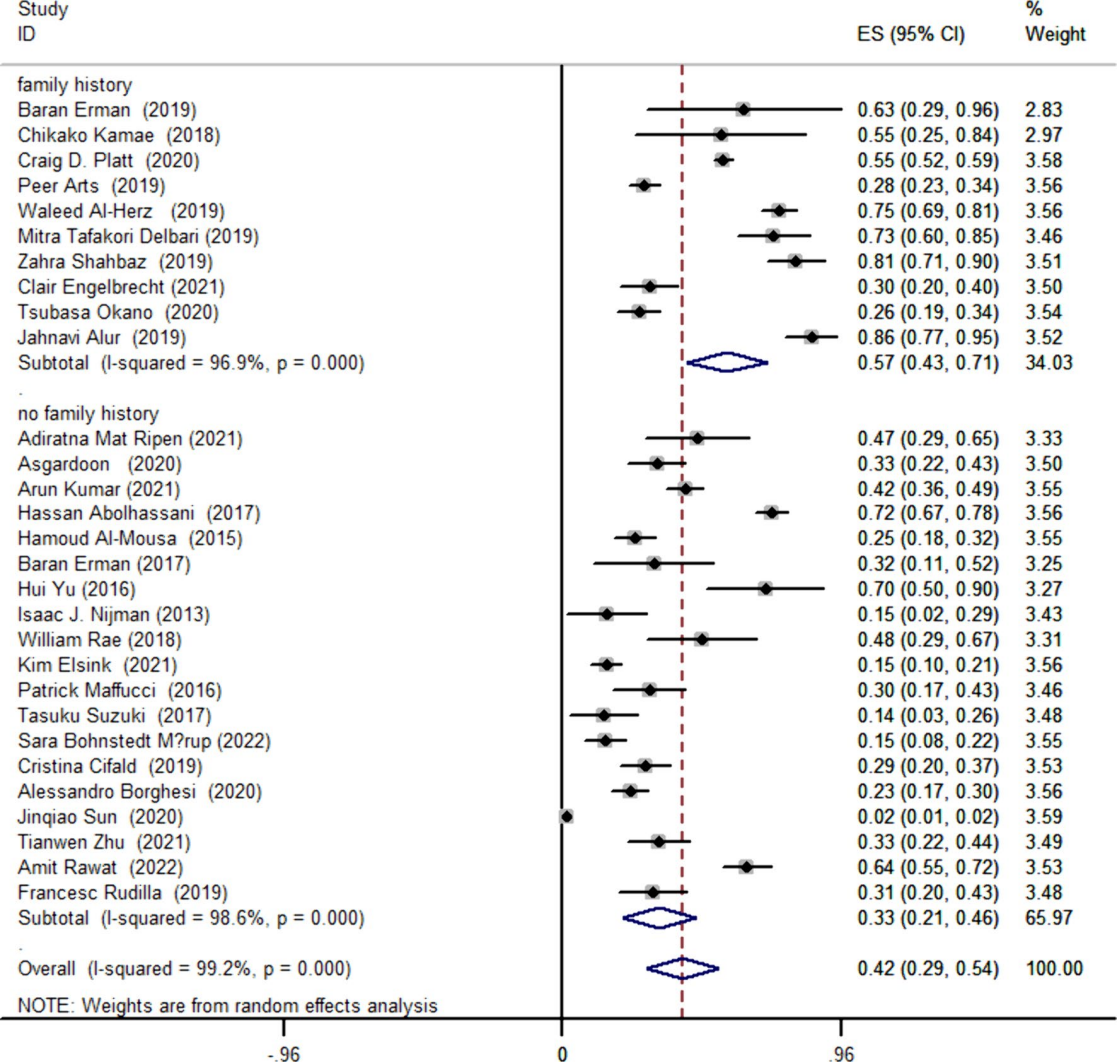
The detection rate of primary immunodeficiency diseases in different populations

**Fig. 4:**
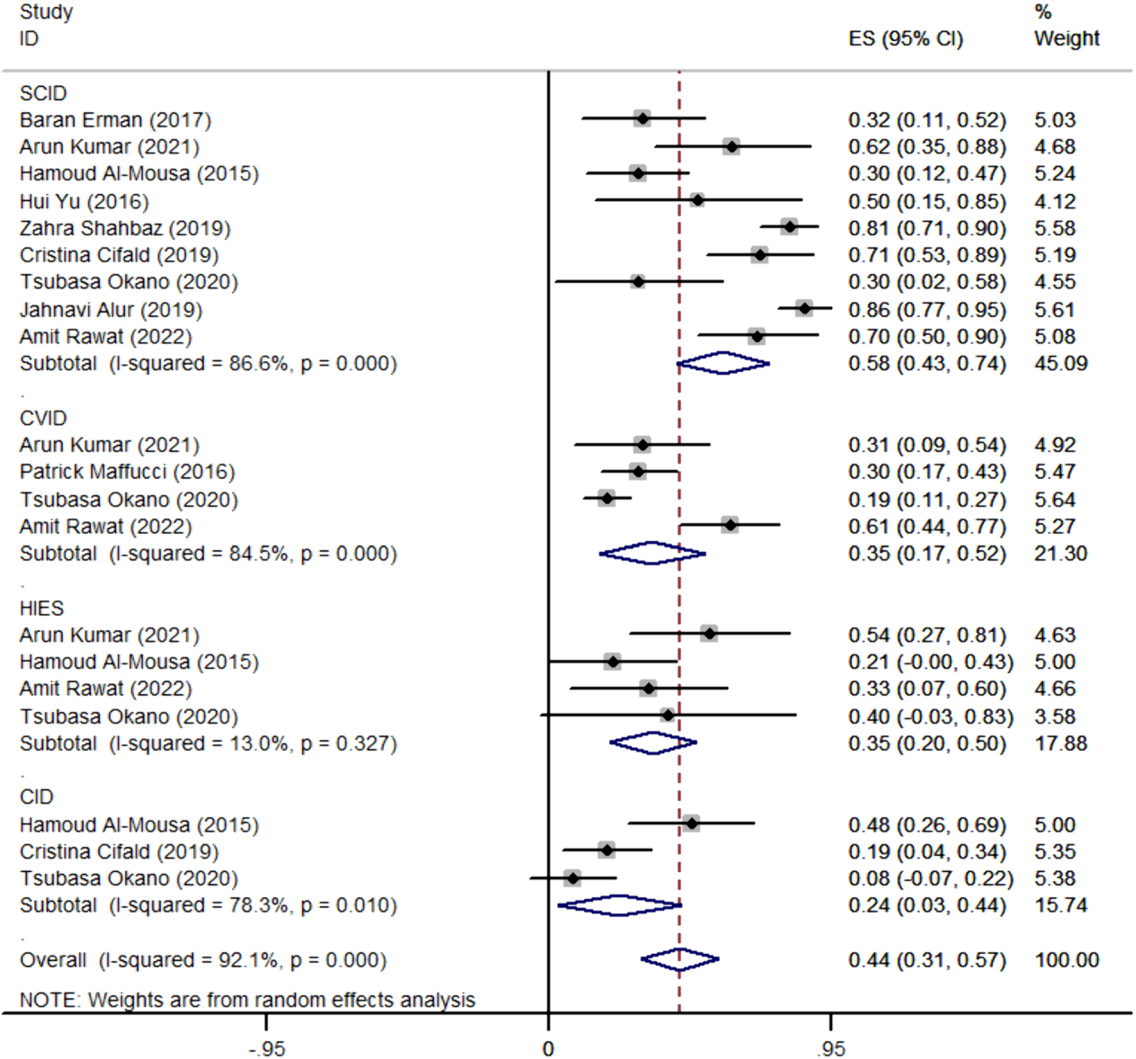
The detection rate of different types of primary immunodeficiency diseases

This study involved 5847 cases, with 1603 cases diagnosed as positive for NGS. Utilizing a random-effects model, the pooled positive detection rate was 42% (95% CI 0.29–0.54, *P* < 0.001) (Fig. 2). Additionally, we conducted subgroup analyses based on family history and different disease types. In patients with a family history, the detection rate could reach 58% (95% CI 0.43–0.71, *P* < 0.001), while in patients without a family history, the detection rate was 33% (95% CI 0.21–0.46, *P* < 0.001) (Fig. 3). We selected diseases mentioned in a larger number of articles as grouping criteria, including Severe Combined Immunodeficiency (SCID), Common Variable Immunodeficiency (CVID), Hyper IgE syndrome (HIES), and Combined Immunodeficiency (CID). The overall detection rate for these four diseases was 44% (95% CI 0.31–0.57, *P* < 0.001), with a detection rate of 58% (95% CI 0.43–0.74, *P* < 0.001) for SCID, 35% (95% CI 0.17–0.52, *P* < 0.001) for CVID, 35% (95% CI 0.20–0.50, *P* < 0.001) for HIES, and 24% (95% CI 0.03–0.44, *P* = 0.026) for CID (Fig. 4).

### Genetic landscape of PID-related genes

**Fig. 5:**
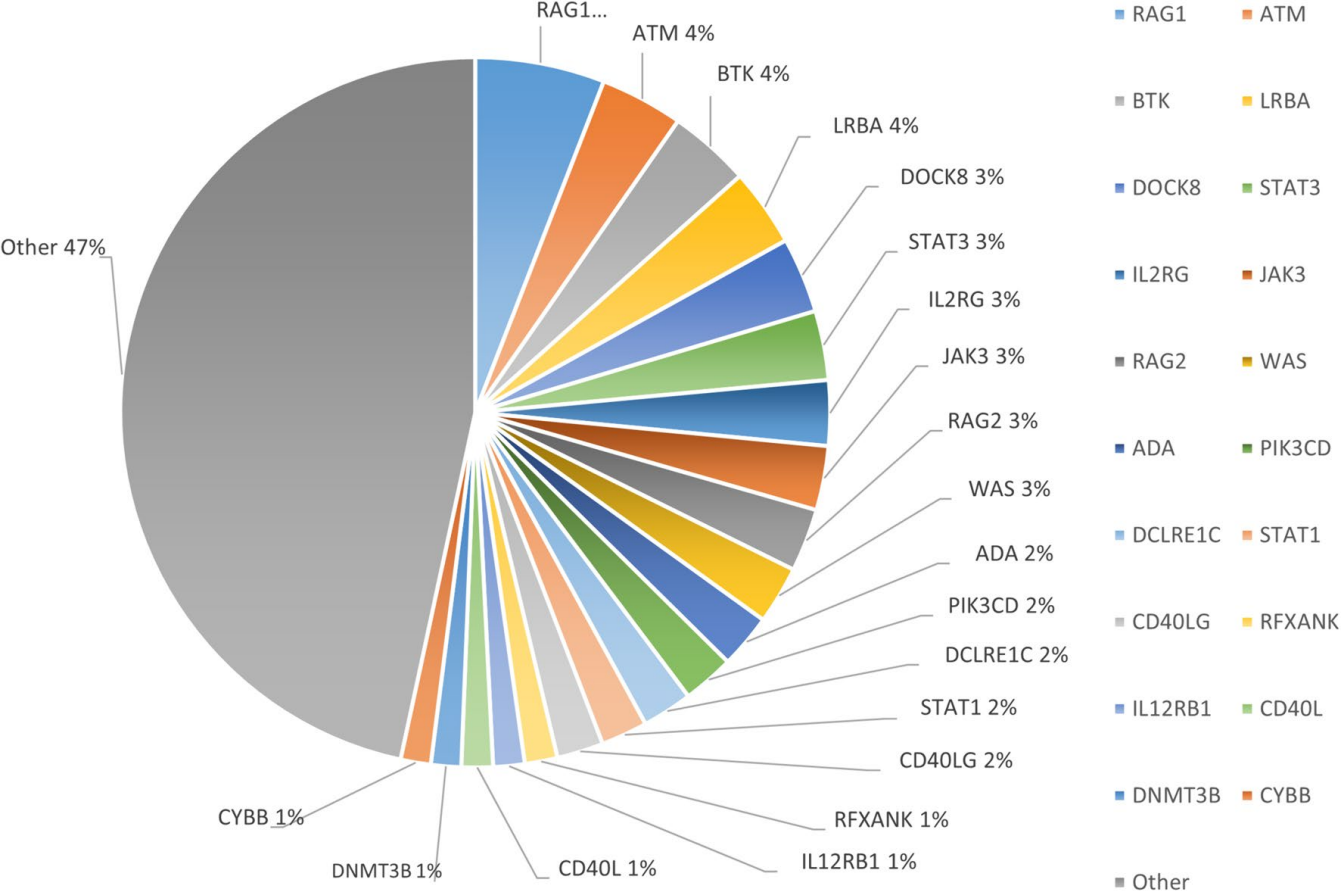
Gene types

A total of 253 PID-related genes were examined, with notable pathogenic mutations mainly involving RAG1, ATM, BTK, LRBA, DOCK8, STAT3, IL2RG, JAK3, RAG2, and WAS, each accounting for more than 3% of the cases, with RAG1 accounting for 6% (Fig. 5). Notably, 34 genes were not included in the updated 2022 IUIS gene list, and additional information on these genes can be found in Table 2.

**Table 2:** The specific information of genes that were not included in the updated 2022 IUIS gene list

Gene list	Locus name	OMIM number	Location	Phenotype	Phenotype MIM number	Inheritance
CD40L	CD40 LIGAND	300386	X:136,648,158-136,660,390	Immunodeficiency, X-linked, with hyper-IgM	308230	XLR
ACIDA	ACIDIC NUCLEAR PHOSPHOPROTEIN 32 FAMILY, MEMBER A	600832	15:68,778,535-68,820,895	NA	NA	AD
RECQL4	DNA helicase, RecQ-like 4	603780	8:144,511,288 8q24.3	Baller-Gerold syndrome; RAPADILINO syndrome; Rothmund-Thomson syndrome, type 2	218600; 266280; 268400	AR; AR; AR
SLC37A4	Solute carrier family 37 (glucose-6-phosphate transporter), member 4	602671	11:119,024,112 11q23.3	Congenital disorder of glycosylation, type Iiw; Glycogen storage disease Ib; Glycogen storage disease Ic	619525 232220 232240	AD; AR; AR
MAVS	Mitochondrial antiviral signaling protein	609676	20:3,846,8320p13	NA	NA	AR
IL7RA	Interleukin-7 receptor	146661	5:35,856,891 5p13.2	Immunodeficiency, severe combined	608971	AR
FASLG	Fas ligand (TNF superfamily, member 6)	134638	1:172,659,103 1q24.3	Lung cancer, Autoimmune lymphoproliferative syndrome, type IB	211980 601859	AD; AD
DHFR	Dihydrofolate reductase	126060	5:80,626,226 5q14.1	Megaloblastic anemia due to dihydrofolate reductase deficiency	613839	AR
TXBP2	TATA BOX-BINDING PROTEIN	600075	6:170,554,369-170,572,859	Spinocerebellar ataxia	607136	AD
TNFSFR13B	Tumor necrosis factor ligand superfamily, member 13B	603969	13:108,269,62913q33.3	NA	NA	AR
TNFSF9	Tumor necrosis factor ligand superfamily, member 9	606182	19:6,531,02619p13.3	NA	NA	NA
TALDO1	Transaldolase-1	602063	11:747,464 11p15.5	Transaldolase deficiency	606003	AR
SP1NK5	Serine protease inhibitor, Kazal type, 5	605010	5:148,063,9805q32	Netherton syndrome	256500	AR
SLC27A4	Solute carrier family 27 (fatty acid transporter), member 4	604194	9:128,340,5279q34.11	Ichthyosis prematurity syndrome	608649	AR
SIX6	SIX homeobox 6	606326	14:60,509,14614q23.1	Optic disc anomalies with retinal and/or macular dystrophy	212550	AR
SH2DA1	SH2 domain protein 1A	300490	X:124,346,563 Xq25	Lymphoproliferative syndrome, X-linked	308240	XLR
RSPH9	Radial spoke head 9 homolog	612648	6:43,645,0366p21.1	Ciliary dyskinesia, primary	612650	AR
RPL5	Ribosomal protein L5	603634	1:92,831,986 1p22.1	Diamond-Blackfan anemia	612561	AD
NFKBID	Nuclear factor kappa-B inhibitor, delta	618887	19:35,887,95219q13.12	NA	NA	NA
NEIL3	Endonuclease VIII-like 3	608934	4:177,309,8744q34.3	NA	NA	NA
NBEAL2	Neurobeachin-like 2	614169	3:46,979,666 3p21.31	Gray platelet syndrome	139090	AR
MYO5B	Myosin VB	606540	18:49,822,789 18q21.1	Cholestasis, progressive familial intrahepatic, Diarrhea , with microvillus atrophy, with or without cholestasis	619868	AR
					251850	AR
LAG3	Lymphocyte activation gene-3	153337	12:6,772,520 12p13.31	NA	NA	NA
				Bleeding disorder, platelet-type, 16, autosomal dominant;	187800	AD
ITGA2B	Integrin, alpha-2b (platelet glycoprotein IIb of IIb/IIIa complex, antigen CD41B)	607759	17:44,372,181 17q21.31	Glanzmann thrombasthenia 1;	273800	AR
HBB IKBKE	Hemoglobin beta Inhibitor of nuclear factor kappa-B kinase, subunit epsilon	141900 605048	11:5,225,46411p15.4 1:206,470,476 1q32.1	Thrombocytopenia, neonatal alloimmune, BAK antigen related	NA	NA
CR2	Complement component (3d/Epstein-Barr virus) receptor-2	120650	1:207,454,328 1q32.2	Immunodeficiency, common variable; Systemic lupus erythematosus	614699	AR
					610927	
CBL	CBL protooncogene	165360	11:119,206,339 11q23.3	Juvenile myelomonocytic leukemia;	607785	AD;
				Noonan syndrome-like disorder with or without juvenile myelomonocytic leukemia	613563	AD
BANK1	B-cell scaffold protein with ankyrin repeats 1	610292	4:101,790,730 4q24	NA	NA	NA
ANK1	Ankyrin-1, erythrocytic	612641	8:41,653,225 8p11.21	Spherocytosis	182900	AD, AR
AHEZL	NA	NA	NA	NA	NA	NA
DCK1	NA	NA	NA	NA	NA	NA
RAG	NA	NA	NA	NA	NA	NA
GATA	NA	NA	NA	NA	NA	NA

### Quality assessment of included studies

**Fig. 6:**
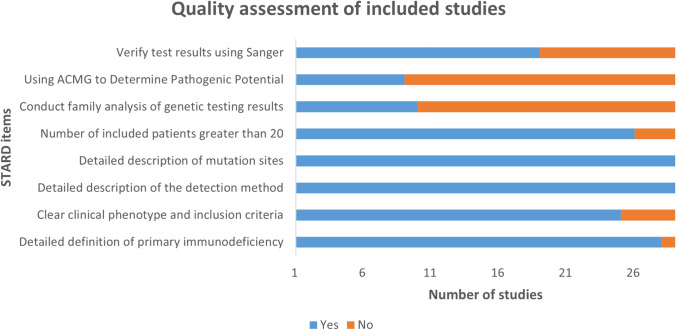
Quality assessment of included studies

Quality assessment utilizing a modified version of the Standards for Reporting of Diagnostic Accuracy (STARD) specific to this project indicated high-quality studies (Fig. 6). As the analysis is a single-group rate analysis with descriptive results, publication bias assessment was deemed unnecessary.

## Discussion

Primary Immunodeficiency Diseases (PIDs) represent a class of inherent immunodeficiency disorders, attributed to genetic mutations impacting distinct facets of the immune system. This diverse group manifests through recurrent infections, autoimmunity, autoinflammation, hypersensitivity reactions, and malignancies [9]. PIDs exhibit genetic heterogeneity, with different mutations in the same gene yielding varied clinical and immune phenotypes, while distinct gene mutations may produce similar clinical outcomes [42]. The broad clinical spectrum, coupled with genetic heterogeneity and pleiotropy, renders the diagnosis and treatment of PIDs challenging. A precise molecular diagnosis is crucial for accurate recognition and tailored intervention in clinical practice.

Our study reveals a positive detection rate of 42% (95% CI 0.29–0.54, *P* < 0.001) for Next-Generation Sequencing (NGS) in patients displaying clinical symptoms associated with PIDs. Previous literature reports a variable detection rate for PIDs, ranging from 15 to 80%. In our analysis of 5847 cases, the observed 42% detection rate falls within an intermediate range. Three key factors contribute to this result: Firstly, the expansive array of clinical manifestations related to PIDs has led to a surge in suspected cases. Secondly, the limited nucleotide coverage of NGS diminishes the potential for detecting pathogenic mutations. Additionally, our study excludes cases diagnosed through Copy Number Variation (CNV) detection [17, 35, 44], a method reported in the literature to offer a substantial number of additional genetic diagnoses [35]. Due to the complexity of these factors, determining an average diagnostic rate poses challenges. Enhancing the accuracy of this rate involves the meticulous collection of comprehensive clinical data.

Our subgroup analysis, differentiating patients based on the presence or absence of a family history and various disease types, yielded intriguing insights. Notably, the detection rate among patients with a family history stands at an impressive 58% (95% CI 0.43–0.71, *P* < 0.001), while their counterparts without a family history exhibit a lower detection rate of 33% (95% CI 0.21–0.46, *P* < 0.001). This stark contrast underscores the substantial impact of familial factors on the detection rate of Primary Immunodeficiency Diseases (PIDs). Supporting this finding, existing literature posits that a majority of PIDs follow an autosomal recessive (AR) inheritance pattern. Identifying individuals with a singular clinical and immune phenotype within consanguineous families emerges as a pivotal strategy for uncovering novel pathogenic genes[2]. Our study, incorporating data from 10 relevant articles [2, 4, 5, 13, 15, 18, 32, 38, 25, 26] involving patients with a family history, aligns with this genetic landscape. Among these, 2 cases inherited PIDs as autosomal recessive (AR) traits, 1 as a combination of AR and X-linked (XL) traits, 6 as a blend of AR, autosomal dominant (AD), and XL traits, and 1 did not specify the genetic features. This consistency with prior research underscores the crucial role of families with consanguinity in unraveling intricate phenotypes associated with PIDs. Exploring consanguineous relationships further reveals their potential to unveil new pathogenic genes, delineate genetic patterns of known genes, identify novel clinical phenotypes, and elucidate novel manifestations linked to established PID-causing genes. Within this context, Next-Generation Sequencing (NGS) emerges as a promising tool for eugenics in families with consanguinity. Recommendations for NGS testing in families, especially post-pregnancy following a PID diagnosis, offer valuable insights into gauging the likelihood of disease inheritance in subsequent generations.

Our investigation underscores distinctive detection rates across various primary immunodeficiency diseases (PIDs). Severe Combined Immunodeficiency (SCID) emerges with the highest detection rate, followed by Common Variable Immunodeficiency (CVID), Hyper-IgE Syndrome (HIES), and Combined Immunodeficiency (CID). Clinical manifestations of Severe Combined Immunodeficiency (SCID) denote a complex spectrum of disorders characterized by impaired T lymphocyte development, impacting the quantity and functionality of B cells and NK cells [34]. SCID stands out as a profoundly severe subset within the broader PID landscape, marked by early-onset dermatitis, dermal complications, persistent enteritis, pneumonia, oral candidiasis, and other distinctive manifestations [43]. Notably, more than 50% of SCID patients have been reported to harbor mutations in the RAG1 or RAG2 genes [33, 41]. In the absence of immune reconstitution, the survival prognosis for SCID patients beyond 6–12 months is exceedingly poor. Nonetheless, this patient cohort commonly demonstrates a favorable response to allogeneic hematopoietic stem cell transplantation (HSCT) [41]. Hence, early disease recognition and expeditious intervention hold the potential to substantially augment patient survival rates. Similarly, diseases such as Common Variable Immunodeficiency (CVID), Hyper-IgE Syndrome (HIES), and Combined Immunodeficiency (CID) constitute prevalent categories within the PID spectrum. The extensive range of diseases within PIDs manifests shared clinical manifestations, necessitating timely and precise identification of disease types. Next-generation sequencing (NGS) emerges as a critical tool for optimizing treatment effectiveness and providing early benefits to patients. However, it is crucial to acknowledge that the literature data incorporated into our study, while informative, is not derived from large-scale studies. The potential introduction of slight errors in the results emphasizes the need for more extensive clinical data to validate our conclusions thoroughly. Large-scale studies will enhance the robustness of our findings, contributing to a more comprehensive understanding of the disease-specific landscape within PIDs.

A critical facet of our study involves scrutinizing primary immunodeficiency diseases (PIDs)-related genes updated by the International Union of Immunological Societies (IUIS) in 2022 [40]. Our analysis identified 34 genes absent from the IUIS list, prompting a deeper investigation. Consulting the Online Mendelian Inheritance in Man (OMIM) database revealed six genes with significant relevance to PIDs' clinical manifestations. CD40L's potential involvement in Immunodeficiency and hyper-IgM, RECQL4's associations with Baller-Gerold syndrome, RAPADILINO syndrome, and Rothmund-Thomson syndrome, and IL7RA's connection to severe combined immunodeficiency exemplify the intricate genetic landscape. Additionally, SP1NK5, ITGA2B, and CR2 exhibit diverse implications, linking to Netherton syndrome, bleeding disorders, and common variable immunodeficiency, respectively. Despite the potential of these genes as targets for clinical testing, their validation is hindered by limited clinical data, underscoring the need for comprehensive validation efforts.

This article still has certain limitations. Firstly, the meta-analysis included a total of 29 articles, and some of the sequencing results in these articles were not validated using Sanger sequencing. Secondly, we have excluded positive cases determined through CNVs detection when entering the data, which may provide accurate information. Thirdly, some of the included literature had a small number of patients tested (< 20), which could affect the pooled results and lead to minor errors. More clinical sequencing data is required to validate the relevant conclusions.

## Conclusion

The application of NGS in suspected PID patients can provide significant diagnostic results, especially in patients with a family history. Meanwhile, NGS performs excellently in accurately diagnosing disease types, and early identification of disease types can benefit patients in treatment.

## Data Availability

The original contributions presented in the study are included in the article/Supplementary Material. Further inquiries can be directed to the corresponding author.
